# Supplementary material for: Skimming for barcodes: rapid production of mitochondrial genome and nuclear ribosomal repeat reference markers through shallow shotgun sequencing
Source: PeerJ. 2022 Aug 5;10:e13790. doi: 10.7717/peerj.13790 (PMC9359134; doi:10.7717/peerj.13790)
Supplement: Table S1 — The sequences from GenBank were accessed March 2022. Sequences were defined as having valid associated specimens when the/specimen_voucher qualifier was present and conformed to valid NCBI BioCollections standard institution and (where included) collection codes. [file peerj-10-13790-s001.docx]

| **Marker** | **Species** | **Total sequences** | **Total sequences with specimen** | **Total sequences with specimen from Hawai‘i** |
| --- | --- | --- | --- | --- |
| 12S | *Gymnura altavela* | 4 | 0 | N/A |
|  | *Gymnothorax fimbriatus* | 4 | 1 | 0 |
|  | *Gymnothorax undulatus* | 4 | 0 | 0 |
|  | *Saurida nebulosa* | 2 | 0 | 0 |
|  | *Tylosurus crocodilus* | 7 | 0 | 0 |
|  | *Myripristis vittata* | 2 | 0 | 0 |
|  | *Neoniphon sammara* | 5 | 2 | 0 |
|  | *Brosme brosme* | 5 | 1 | N/A |
|  | *Scomberoides lysan* | 5 | 1 | 0 |
|  | *Forcipiger flavissimus* | 7 | 1 | 0 |
|  | *Ostracion whitleyi* | 2 | 0 | 0 |
|  | *Canthigaster amboinensis* | 3 | 1 | 0 |
| 16S | *Gymnura altavela* | 2 | 0 | N/A |
|  | *Gymnothorax fimbriatus* | 6 | 4 | 0 |
|  | *Gymnothorax undulatus* | 5 | 3 | 0 |
|  | *Saurida nebulosa* | 2 | 0 | 0 |
|  | *Tylosurus crocodilus* | 4 | 0 | 0 |
|  | *Myripristis vittata* | 1 | 0 | 0 |
|  | *Neoniphon sammara* | 1 | 0 | 0 |
|  | *Brosme brosme* | 4 | 2 | N/A |
|  | *Scomberoides lysan* | 5 | 0 | 0 |
|  | *Forcipiger flavissimus* | 9 | 4 | 0 |
|  | *Ostracion whitleyi* | 1 | 0 | 0 |
|  | *Canthigaster amboinensis* | 2 | 0 | 0 |
| COI | *Gymnura altavela* | 38 | 8 | N/A |
|  | *Gymnothorax fimbriatus* | 8 | 4 | 0 |
|  | *Gymnothorax undulatus* | 69 | 13 | 1 |
|  | *Saurida nebulosa* | 12 | 10 | 3 |
|  | *Tylosurus crocodilus* | 32 | 22 | 1 |
|  | *Myripristis vittata* | 5 | 3 | 0 |
|  | *Neoniphon sammara* | 61 | 24 | 0 |
|  | *Brosme brosme* | 18 | 14 | N/A |
|  | *Scomberoides lysan* | 35 | 22 | 2 |
|  | *Forcipiger flavissimus* | 36 | 26 | 1 |
|  | *Ostracion whitleyi* | 4 | 2 | 0 |
|  | *Canthigaster amboinensis* | 13 | 11 | 1 |
