# Supplementary material for: Skimming for barcodes: rapid production of mitochondrial genome and nuclear ribosomal repeat reference markers through shallow shotgun sequencing
Source: PeerJ. 2022 Aug 5;10:e13790. doi: 10.7717/peerj.13790 (PMC9359134; doi:10.7717/peerj.13790)
Supplement: Table S2 [file peerj-10-13790-s002.docx]

| **Species** | **Accession Number** |
| --- | --- |
| *Apolemichthys armitagei* | KU244245 |
| *Apolemichthys griffisi* | KU363798 |
| *Apolemichthys kingi* | KU244239 |
| *Centropyge acanthops* | KU356792 |
| *Centropyge aurantia* | KU356780 |
| *Centropyge deborae* | KU356786 |
| *Centropyge eibli* | KU356788 |
| *Centropyge flavicauda* | KU356787 |
| *Centropyge flavissima* | KU356782 |
| *Centropyge flavissima* | KU363797 |
| *Centropyge heraldi* | KU356783 |
| *Centropyge interrupta* | KU356784 |
| *Centropyge joculator* | KU356785 |
| *Paracentropyge multifasciata* | KU356779 |
| *Centropyge nox* | KU356790 |
| *Centropyge venusta* | KU356781 |
| *Genicanthus lamarck* | KU356791 |
| *Genicanthus melanospilos* | KU356789 |
| *Genicanthus semifasciatus* | KU244252 |
| *Holacanthus africanus* | KU244247 |
| *Holacanthus tricolor* | KU356793 |
