# Supplementary figures and images for: Skimming for barcodes: rapid production of mitochondrial genome and nuclear ribosomal repeat reference markers through shallow shotgun sequencing
Source: PeerJ. 2022 Aug 5;10:e13790. doi: 10.7717/peerj.13790 (PMC9359134; doi:10.7717/peerj.13790)

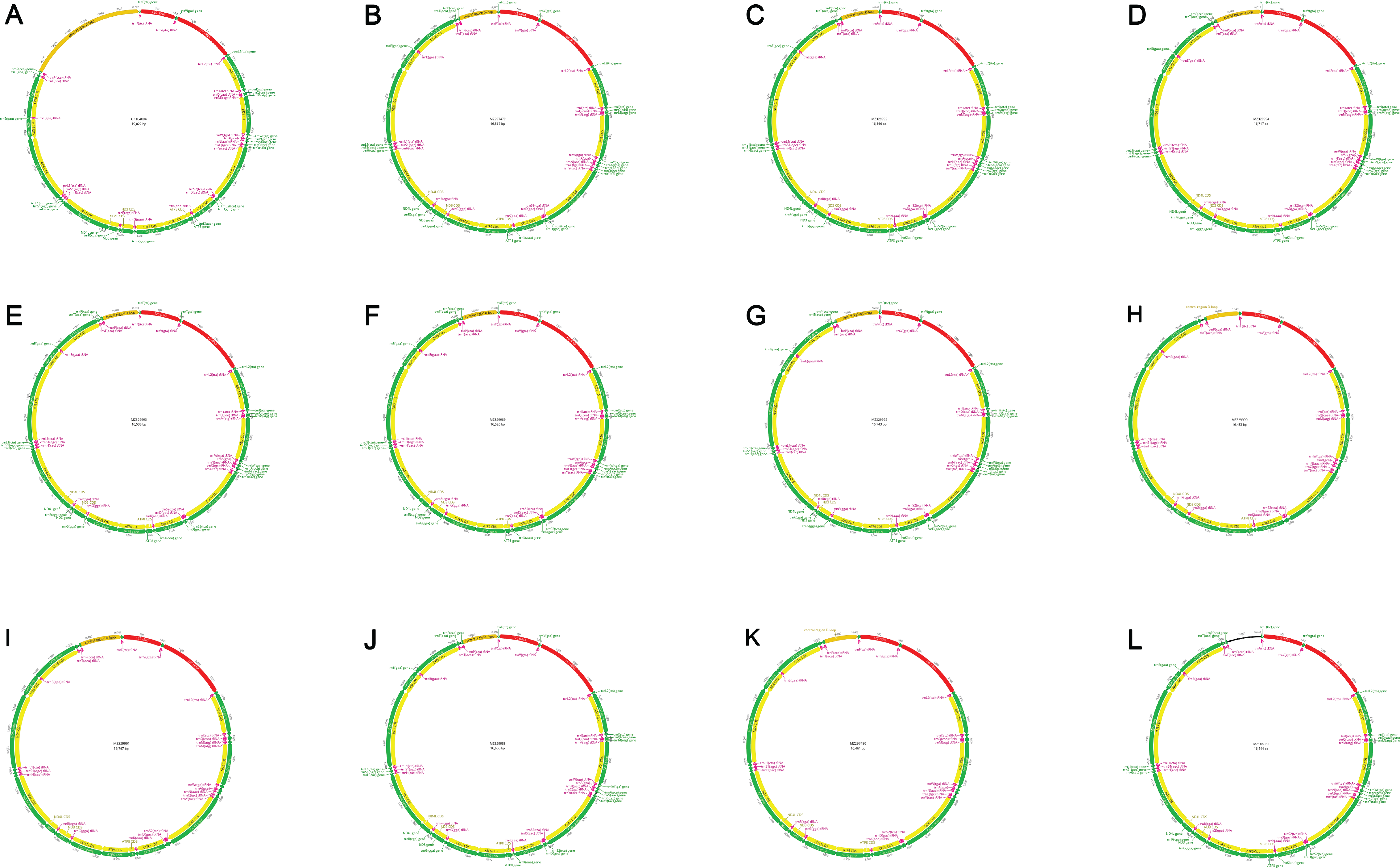

Supplement: Figure S1 — (A)Gymnura altavela. (B) Gymnothorax fimbriatus. (C) Gymnothorax undulatus. (D) Saurida nebulosa. (E) Tylosurus crocodilus. (F) Myripristis vittata. (G) Neoniphon sammara. (H) Brosme brosme. (I) Scomberoides lysan. (J) Forcipiger flavissimus. (K) Ostracion whitleyi. (L) Canthigaster amboinensis. [file peerj-10-13790-s006.png]

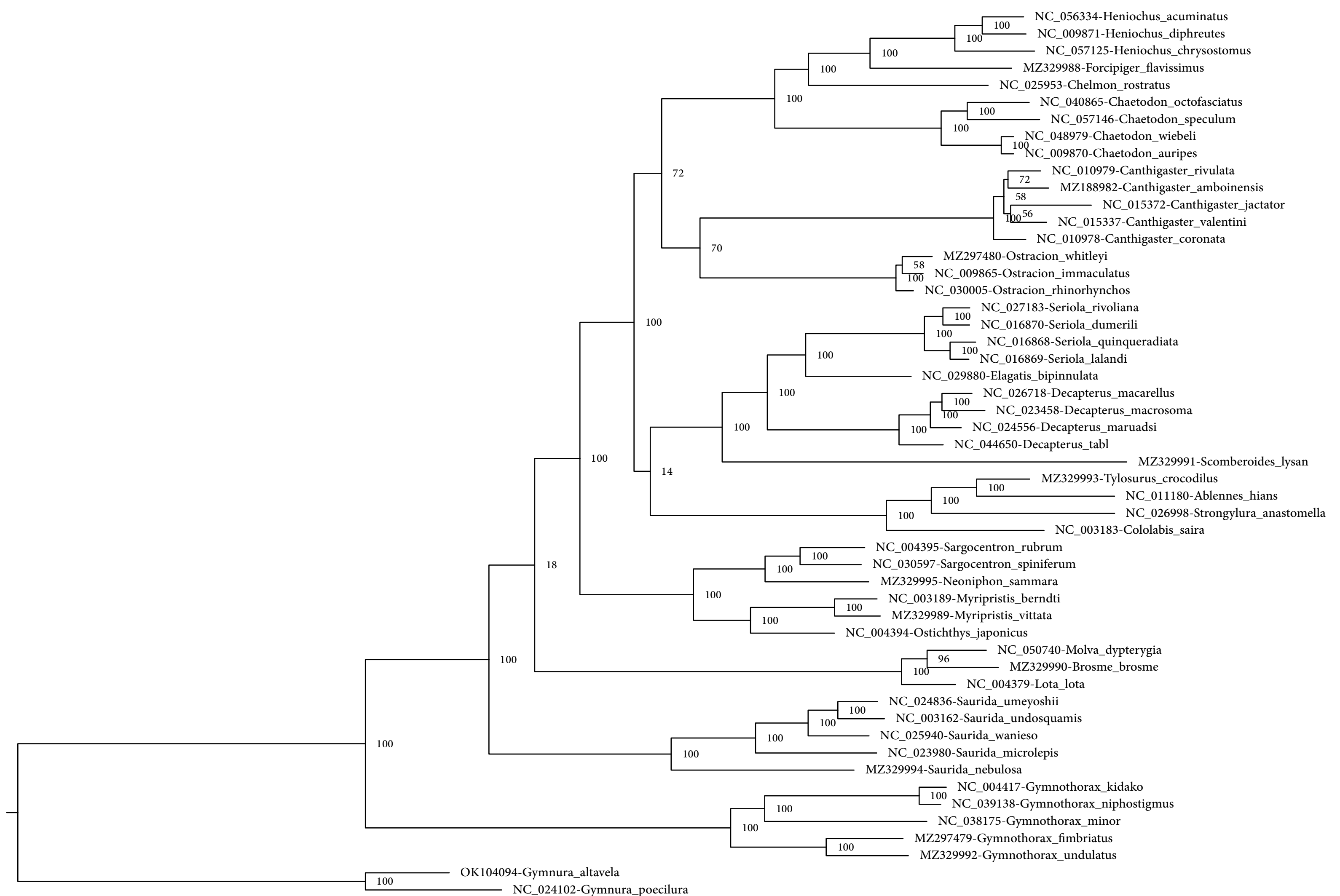

0.6

Supplement: Figure S2 [file peerj-10-13790-s007.pdf]

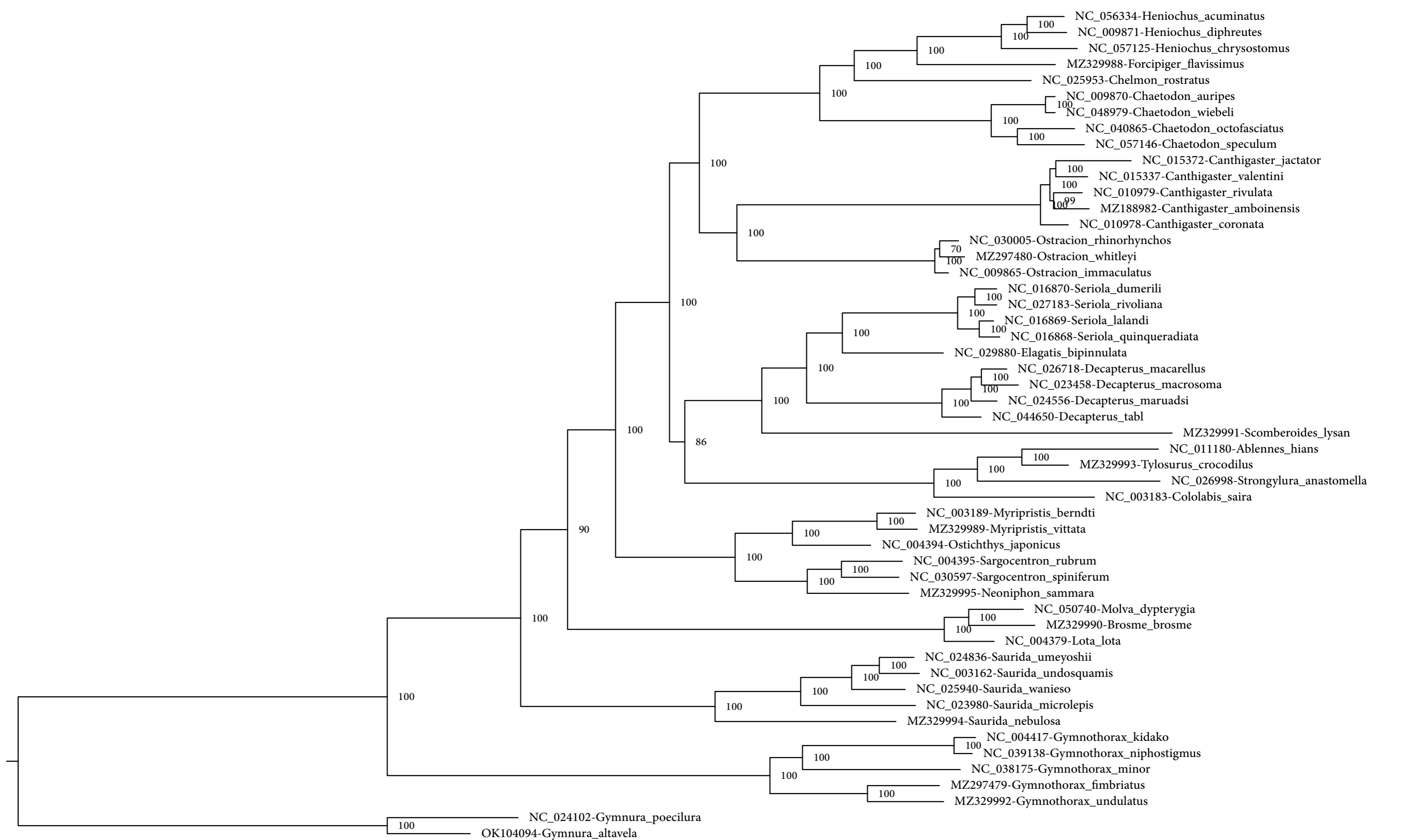

0.4

Supplement: Figure S3 [file peerj-10-13790-s008.pdf]
